# A132 A COLON-SPECIFIC SELECTIVE RESECTION ALGORITHM FOR LARGE NON-PEDUNCULATED POLYPS OPTIMIZES EFFICIENCY WITH HIGH TECHNICAL AND CLINICAL SUCCESS: A PROSPECTIVE COHORT STUDY

**DOI:** 10.1093/jcag/gwad061.132

**Published:** 2024-02-14

**Authors:** S X Jiang, A Zarrin, A Walia, C Galorport, R Enns, E Lam, N Shahidi

**Affiliations:** The University of British Columbia Faculty of Medicine, Vancouver, BC, Canada; The University of British Columbia Faculty of Medicine, Vancouver, BC, Canada; St. Paul’s Hospital, Division of Gastroenterology, University of British Columbia, Vancouver, BC, Canada; St. Paul’s Hospital, Division of Gastroenterology, University of British Columbia, Vancouver, BC, Canada; St. Paul’s Hospital, Division of Gastroenterology, University of British Columbia, Vancouver, BC, Canada; St. Paul’s Hospital, Division of Gastroenterology, University of British Columbia, Vancouver, BC, Canada; St. Paul’s Hospital, Division of Gastroenterology, University of British Columbia, Vancouver, BC, Canada

## Abstract

**Background:**

Endoscopic resection is now the first line treatment strategy for most large (≥20 mm) non-pedunculated colonic polyps (LNPCPs); which includes endoscopic mucosal resection (EMR), cold snare resection (CSR), and endoscopic submucosal dissection (ESD). A selective resection algorithm incorporating EMR and ESD is cost effective and optimizes oncologic outcomes in the rectum. However, a focused evaluation for colonic lesions has not been described.

**Aims:**

Assess the performance of a colon-specific selective resection algorithm for LNPCPs.

**Methods:**

Consecutive patients ampersand:003E 18 years of age who underwent endoscopic resection for a LNPCP were enrolled in a prospective single center observation cohort study (clinicaltrials.gov ID: NCT05402696). Modality selection was determined by optical evaluation using the Paris and the Japan NBI Expert Team (JNET) classifications: 1) JNET I – CSR; 2) JNET IIA – EMR; 3) JNET IIB or Paris 0-IIC morphology – en bloc resection (EMR/ESD); 4) JNET III – referral to surgery/multi-disciplinary team review. Algorithm performance was evaluated by technical success (all neoplastic tissue removed at index procedure), procedure-related adverse events, referral to surgery, and recurrence at first surveillance colonoscopy (SC1).

**Results:**

From 06/2022-09/2023, 230 patients underwent 244 procedures for 295 lesions. Median age was 67 years (IQR 61-74 years) and 127 (55.2%) were male. Median lesion size was 30mm (IQR 20-40mm). Cancer was identified in 12 (4.1%) LNPCPs. Based on pre-resection optical evaluation, 204 (69.2%), 86 (29.2%) and 5 (1.7%) LNPCPs underwent EMR, CSR and ESD, respectively. Technical success was 97.1%, 100.0% and 100.0% for EMR, CSR and ESD, respectively (p=0.245). Procedure duration was significantly shorter for CSR (10 min; IQR 8-15 min) compared to EMR (15 min; IQR 10-25 min) and ESD (30 min; IQR 30-60 min) (P ampersand:003C 0.001). There was no significant difference in intra-procedural perforation, clinically significant post-endoscopic resection bleeding, delayed perforation or serositis, with overall frequencies of 3.4%, 5.2%, 0% and 0.4% respectively. Surgery was performed for 15 lesions (11 submucosal invasive cancer (SMIC); 3 synchronous SMIC; 1 technical failure). Of LNPCPs completing SC1, there was 1 recurrence (1.2%) in the CSR group (p=0.366), which was successfully managed endoscopically.

**Conclusions:**

A colon-specific selective resection algorithm optimizes procedural efficiency and the risk-benefit profiles of EMR, CSR and ESD for LNPCPs.

**Funding Agencies:**

None

